# Plasma apolipoprotein E level is associated with the risk of endobronchial biopsy-induced bleeding in patients with lung cancer

**DOI:** 10.1186/s12944-018-0821-6

**Published:** 2018-07-21

**Authors:** Saibin Wang, Qian Ye, Xiaodong Lu

**Affiliations:** 1 Department of Respiratory Medicine, Jinhua Municipal Central Hospital, No. 365, East Renmin Road, Jinhua, Zhejiang Province, 321000 China; 2 Department of Medical Records Quality Management, Jinhua Municipal Central Hospital, No. 365, East Renmin Road, Jinhua, Zhejiang Province, 321000 China; 3 Department of Laboratory Medicine, Jinhua Municipal Central Hospital, No. 365, East Renmin Road, Jinhua, Zhejiang Province, 321000 China

**Keywords:** Apolipoprotein E, Lung cancer, Endobronchial biopsy, Bleeding

## Abstract

**Background:**

Factors affecting the risk of bleeding by bronchoscopic biopsy in patients with lung cancer remain unclear. The levels of plasma apolipoprotein E (ApoE) that may be associated with endobronchial biopsy (EBB)-induced bleeding have never been examined.

**Methods:**

This was a retrospective study using data collected from 615 consecutive patients who had undergone EBB and been diagnosed with primary lung cancer from January 2014 through February 2018. Patients were either classified as the bleeding group (*n* = 214) or the non-bleeding group (*n* = 391) based on the bronchoscopy report. Multiple regression analysis was done to estimate the independent relationship between ApoE levels and EBB-induced bleeding, with an adjustment for potential confounders.

**Results:**

The mean plasma ApoE concentration was higher in the non-bleeding group compared to that in the bleeding group (*P* < 0.05). However, a non-linear relationship with threshold effects was observed between plasma ApoE levels and EBB-induced bleeding in a piecewise linear regression analysis. The risk of EBB-induced bleeding decreased with ApoE concentrations from 3.5 mg/dL up to 5.9 mg/dL (adjusted odds ratio, 0.64; 95% confidence interval, 0.43–0.94); however, the incidence of EBB-induced bleeding increased with ApoE levels above the turning point (ApoE = 5.9 mg/dL).

**Conclusions:**

There was a non-linear association between plasma ApoE levels and the risk of EBB-induced bleeding. Higher plasma ApoE concentrations (> 5.9 mg/dL) are the independent risk factor for hemorrhage during EBB in patients with lung cancer.

## Background

Patients with lung cancer are the main subjects who need bronchoscopy. Currently, bronchoscopic biopsy is indispensable in histopathological diagnosis of endobronchial exophytic lesions [1]. Common biopsy procedures include endobronchial forceps biopsy, cryobiopsy, bronchial brushing, transbronchial lung biopsy and transbronchial needle aspiration [2, 3]. Endobronchial biopsy (EBB) has been used for the diagnosis of lung disease for over 40 years, and is still one of the most widely used biopsy modalities to date [4]. However, hemorrhage is a very common complication for bronchoscopists during EBB. Malignant lesions are more likely subjected to bleed upon biopsy than benign mucosal lesions [5]. The rate of EBB-induced hemorrhage is over 30.0% in patients with lung cancer [6], and massive biopsy-related hemorrhage could be life-threatening [7, 8].

Several risk factors have been proposed for hemorrhage during bronchoscopy, including mechanical ventilation, immunosuppression, thrombocytopenia, anti-coagulant or anti-platelet therapy, severe liver and kidney disease, heart failure, and pulmonary arterial hypertension [9–11]. However, whether these proposed risk factors are in reality associated with hemorrhage during EBB, is still debatable [12, 13]. In fact, most patients who undergo EBB do not deal with the aforementioned risk factors in clinical practice.

Apolipoprotein E (ApoE) plays a pivotal role in lipoprotein metabolism in the human body. During the last few decades, several studies have demonstrated that ApoE is associated with an increased risk of subarachnoid hemorrhage [14], hypertensive cerebral hemorrhage [15], amyloid angiopathy and angiorrhexis [16, 17]. The increased risk of hemorrhage in these disorders, at least in part, may be attributed to ApoE expression.

We hypothesized that plasma ApoE may be associated with the incidence of biopsy-induced bleeding events, and had the potential to be a biomarker for EBB-induced bleeding. The aim of this study was to explore the association of plasma ApoE with the risk of EBB-induced bleeding in patients with lung cancer.

## Methods

### Patient population

Retrospective review was done on medical records of consecutive patients who underwent EBB and were diagnosed with lung cancer at the Jinhua Municipal Central Hospital between January 2014 and February 2018. The collected patients met the following criteria: a. adult patients with endobronchial local exophytic lesions who underwent forceps biopsies; b. patients diagnosed with a primary lung cancer. Patients with the following “proposed risk factors” were excluded from the study, including bleeding tendencies, active bleeding, platelets < 50 × 10^3^/μl, continuous anti-coagulant or/and anti-platelet therapy, severe liver and kidney disease, heart failure, mechanical ventilation, pulmonary arterial hypertension, lung transplant, and immunosuppression.

Patients were classified into two groups: the bleeding group (those who received hemostasis maneuvers during EBB) and the non-bleeding group (those who did not need hemostasis maneuvers, or did not experience hemorrhage during EBB).

The following details were collected: age, sex, systolic blood pressure (SBP), diastolic blood pressure (DBP), location of the lesions (central airways referred to the trachea, left main bronchi, right main bronchi, and right middle bronchus; whereas peripheral bronchi referred to the left and right lobar bronchi), histological types, TNM stage of cancer (stage I and II as an early stage, and stage III and IV as an advanced stage), and comorbidities (chronic obstructive pulmonary disease (COPD), coronary heart disease (CHD), hypertension, and diabetes). Blood tests included ApoE levels, total cholesterol (TC), triglyceride, low density lipoprotein cholesterol (LDLC), high density lipoprotein cholesterol (HDLC), C-reactive protein (CRP), white blood cell counts, neutrophils, hemoglobin, platelet counts, prothrombin time, activated partial thromboplastin time, aspartate aminotransferase (AST), and alanine aminotransferase (ALT). Blood tests were performed on the first visit or admission, three days prior to EBB.

This study was approved by the ethics committee of Jinhua Hospital of Zhejiang University (No. 2017101003). The requirement for informed consent was waived because the data were anonymous.

### Bronchoscopic procedures

Bronchoscopic procedures were performed under general anesthesia. We used Propofol (1.0 mg/kg) for induction and (3.0–6.0 mg/kg/h) for anesthesia maintenance. Remifentanil (5.0–10.0 μg/kg/h) was used for analgesia. Cisatracurium was used for the induction of neuromuscular blockade, as needed. A laryngeal mask airway (LMA) (Well Lead Medical Co., Ltd., Guangzhou, China) was used, and the bronchoscopic procedures were performed via LMA. Patients were ventilated using a closed circuit connected to the ventilator during the bronchoscopy. Fiberoptic bronchoscopy (BF-1 T60, Olympus Corp., Tokyo, Japan) was performed on all patients by two experienced bronchoscopists.

Generally, three to five biopsies were performed in each patient at the same location of the lesion using forceps biopsies [2], but only one biopsy was performed when some endobronchial lesions bled significantly following the first biopsy attempt. Cold water (4 °C physiological saline), diluted adrenalin (1:10000), and argon plasma coagulation (APC) were used for hemostasis.

### Statistical analysis

Descriptive statistics were used to summarize baseline characteristics. Age was indicated as median (range), categorical variables were expressed as a number (percentage), and blood test values were presented as mean ± standard error of mean. Unpaired t-tests, Pearson chi-squared tests or the Fisher’s exact, were tested between two groups comparison, as appropriate. We performed multiple regression analysis to estimate the independent relationship between plasma ApoE concentrations and the risk of EBB-induced bleeding, with an adjustment for potential confounders. A two-piecewise linear regression was used to test the threshold effect of ApoE on EBB-induced bleeding using a smoothing function. All analyses were performed using R (The R Foundation; https://www.r-project.org) software and Empower (X&Y solutions, Inc., Boston, MA; http://www.empowerstats.com). A value of *P* < 0.05 was considered statistically significant.

## Results

Of 615 consecutive patients, 224 (36.4%) hemorrhaged during EBB, and they were subjected to hemostasis maneuvers (cold water, diluted adrenalin or/and APC). No case of severe bleeding was recorded. Baseline characteristics and blood test results are presented in Table 1.

**Table 1:** Baseline Characteristics and Blood tests of the Study Participants

Characteristics	Biopsy-induced bleeding	
	No (n = 391)	Yes (*n* = 224)
Age (y), median (range)	65.00 (32–85)	65.50 (36–82)
Sex, n (%)		
Female	94 (24.04)	41 (18.30)
Man	297 (75.96)	183 (81.70)
Smoking, n (%)		
Never	158 (40.41)	68 (30.36)
Former	56 (14.32)	44 (19.64)
Current	177 (45.27)	112 (50.00)
SBP (mmHg)	132.34 ± 0.98	130.01 ± 1.37
DBP (mmHg)	77.95 ± 0.58	78.60 ± 0.82
Location of lesion, n (%)		
Peripheral bronchi	362 (92.58)	175 (78.12)
Central airway	29 (7.42)	49 (21.88)
Stage, n (%)		
Early	237 (60.61)	98 (44.95)
Advanced	154 (39.39)	120 (55.05)
Histological types, n (%)		
Adenocarcinoma	130 (33.25)	36 (16.07)
Squamous cell carcinoma	175 (44.76)	134 (59.82)
SCLC	67 (17.14)	41 (18.30)
Others	19 (4.86)	13 (5.80)
COPD, n (%)		
No	365 (93.35)	208 (92.86)
Yes	26 (6.65)	16 (7.14)
Hypertension, n (%)		
No	297 (75.96)	166 (74.11)
Yes	94 (24.04)	58 (25.89)
Diabetes, n (%)		
No	370 (94.63)	214 (95.54)
Yes	21 (5.37)	10 (4.46)
CHD, n (%)		
No	379 (96.93)	216 (96.43)
Yes	12 (3.07)	8 (3.57)
Triglyceride (mmol/L)	1.24 ± 0.03	1.15 ± 0.05
TC (mmol/L)	4.19 ± 0.06	4.10 ± 0.06
HDLC (mmol/L)	1.18 ± 0.02	1.12 ± 0.02
LDLC (mmol/L)	2.84 ± 0.04	2.77 ± 0.05
Apo E (mg/dL)	3.90 ± 0.07	3.65 ± 0.10
Apo B (g/L)	1.02 ± 0.02	0.99 ± 0.02
Homocysteine (μmol/L)	15.05 ± 0.43	20.48 ± 5.23
WBC (× 10^9^/L)	7.31 ± 0.16	7.62 ± 0.22
Neutrophils (×10^9^/L)	5.23 ± 0.16	5.70 ± 0.22
Hemoglobin (g/L)	127.66 ± 1.69	126.96 ± 1.71
platelets (× 10^9^/L)	229.10 ± 4.34	241.23 ± 6.63
CRP (mg/L)	21.81 ± 1.73	29.81 ± 2.61
PT (S)	16.04 ± 1.22	13.90 ± 0.74
APTT (S)	38.83 ± 1.38	35.58 ± 1.15
ALT (IU/L)	22.27 ± 0.91	21.48 ± 1.39
AST (IU/L)	26.54 ± 0.73	26.52 ± 1.20

*SBP* systolic blood pressure, *DBP* diastolic blood pressure, *SCLC* small-cell lung carcinoma, *COPD* Chronic obstructive pulmonary disease, *CHD* coronary heart disease, *TC* total cholesterol, *HDLC* high density lipoprotein cholesterol, *LDLC* low density lipoprotein cholesterol, *Apo* apolipoprotein, *WBC* white blood cell, *CRP* C-reactive protein, *PT* prothrombin time, *APTT* activated partial thromboplastin time, *ALT* alanine aminotransferase, *AST* aspartate aminotransferase

The plasma ApoE levels were higher in the non-bleeding group compared to those in the bleeding group (Table 2, *P* < 0.05). In addition, the location of the lesion, histological types, stage, smoking history, HDLC, and CRP level positively correlated with EBB-induced hemorrhage as assessed by univariate analysis (Table 2).

**Table 2:** Univariate Analysis of Possible Influencing Factors of the Risk of Biopsy-induced Bleeding

Variables	Biopsy-induced Bleeding	
	OR (95% CI)	*P* value
Age (y), median (range)	1.01 (0.99, 1.03)	0.1909
Sex, n (%)		
Female	Ref.	
Man	1.41 (0.94, 2.13)	0.0990
Smoking, n (%)		
Never	Ref.	
Former	1.83 (1.12, 2.97)	0.0153
Current	1.47 (1.02, 2.13)	0.0411
SBP (mmHg)	0.99 (0.99, 1.00)	0.1595
DBP (mmHg)	1.00 (0.99, 1.02)	0.5100
Location of lesion, n (%)		
Peripheral bronchi	Ref.	
Central airway	3.50 (2.13, 5.72)	< 0.0001
Stage, n (%)		
Early	Ref.	
Advanced	1.88 (1.35, 2.63)	0.0002
Histological types, n (%)		
Adenocarcinoma	Ref.	
Squamous cell carcinoma	2.77 (1.79, 4.26)	< 0.0001
SCLC	2.21 (1.29, 3.78)	0.0037
Others	2.47 (1.11, 5.48)	0.0260
COPD, n (%)		
No	Ref.	
Yes	1.08 (0.57, 2.06)	0.8155
Hypertension, n (%)		
No	Ref.	
Yes	1.10 (0.76, 1.61)	0.6085
Diabetes, n (%)		
No	Ref.	
Yes	0.82 (0.38, 1.78)	0.6214
CHD, n (%)		
No	Ref.	
Yes	1.17 (0.47, 2.91)	0.7356
Triglyceride (mmol/L)	0.80 (0.61, 1.05)	0.1084
TC (mmol/L)	0.91 (0.77, 1.07)	0.2707
HDLC (mmol/L)	0.52 (0.31, 0.88)	0.0146
LDLC (mmol/L)	0.89 (0.71, 1.11)	0.2856
Apo E (mg/dL)	0.89 (0.80, 1.00)	0.0488
Apo B (g/L)	0.76 (0.46, 1.27)	0.2949
Homocysteine (μmol/L)	1.00 (0.99, 1.01)	0.4514
WBC (×109/L)	1.03 (0.98, 1.08)	0.2423
Neutrophils (×109/L)	1.04 (0.99, 1.10)	0.0856
Hemoglobin (g/L)	1.00 (0.99, 1.00)	0.7850
platelets (× 109/L)	1.00 (1.00, 1.00)	0.1130
CRP (mg/L)	1.01 (1.00, 1.01)	0.0093
PT (S)	0.99 (0.98, 1.01)	0.2445
APTT (S)	0.99 (0.98, 1.00)	0.1430
ALT (IU/L)	1.00 (0.99, 1.01)	0.6193
AST (IU/L)	1.00 (0.99, 1.01)	0.9870

*SBP* systolic blood pressure, *DBP* diastolic blood pressure, *SCLC* small-cell lung carcinoma, *COPD* Chronic obstructive pulmonary disease, *CHD* coronary heart disease, *TC* total cholesterol, *HDLC* high density lipoprotein cholesterol, *LDLC* low density lipoprotein cholesterol, *Apo* apolipoprotein, *WBC* white blood cell, *CRP* C-reactive protein, *PT* prothrombin time, *APTT* activated partial thromboplastin time, *ALT* alanine aminotransferase, *AST* aspartate aminotransferase

Table 3 shows the association between plasma ApoE and the risk of EBB-induced bleeding after adjusting for the location of the lesion, histological types, stage, smoking history, HDLC, and CRP level (adjust I), and on combined adjustment of risk factors judged by clinical significance (age, sex, diabetes, hypertension, COPD, CHD, SBP, DBP, TC, triglyceride, LDLC, ALT, and AST) (adjust II). We found that middle levels of ApoE (3.5–5.9 mg/dL) associated well with a decreased risk of EBB-induced bleeding when compared to lower levels (< 3.5 mg/L) (odds ratio [OR], 0.64; 95% confidence interval [CI], 0.43–0.94; *P* < 0.05); however higher levels of ApoE (≧ 6.0 mg/L) did not associate with a decreased incidence of EBB-induced bleeding when compared to low level of ApoE (< 3.5 mg/L) (OR, 0.85; 95% CI, 0.41–1.76; *P* > 0.05).

**Table 3:** Multivariate Regression Analysis of ApoE With the Risk of Biopsy-induced Bleeding

ApoE (mg/dL)	Biopsy-induced bleeding OR (95% CI) *P*-value		
	Non-adjust	Adjust I^a^	Adjust II^b^
< 3.5	Ref.	Ref.	Ref.
3.6–5.9	0.59 (0.42, 0.84) 0.0029	0.63 (0.43, 0.91) 0.0140	0.64 (0.43, 0.94) 0.0228
≧ 6.0	0.74 (0.39, 1.39) 0.3487	0.86 (0.44, 1.71) 0.6731	0.85 (0.41, 1.76) 0.6634

^a^Adjust I adjust for: HDLC, smoking, location of lesion, histological types, stage, and CRP

^b^Adjust II adjust for: Sex, Age, SBP, DBP, smoking, TC, triglyceride, HDLC, LDLC, location of lesion, histological types, stage, diabetes, hypertension, COPD, CHD, ALT, AST, and CRP

*Apo* apolipoprotein, *HDLC* high density lipoprotein cholesterol, *CRP* C-reactive protein, *SBP* systolic blood pressure, *DBP* diastolic blood pressure, *TC* total cholesterol, *LDLC* low density lipoprotein cholesterol, *COPD* Chronic obstructive pulmonary disease, *CHD* coronary heart disease, *ALT* alanine aminotransferase, *AST* aspartate aminotransferase

**Fig. 1:**
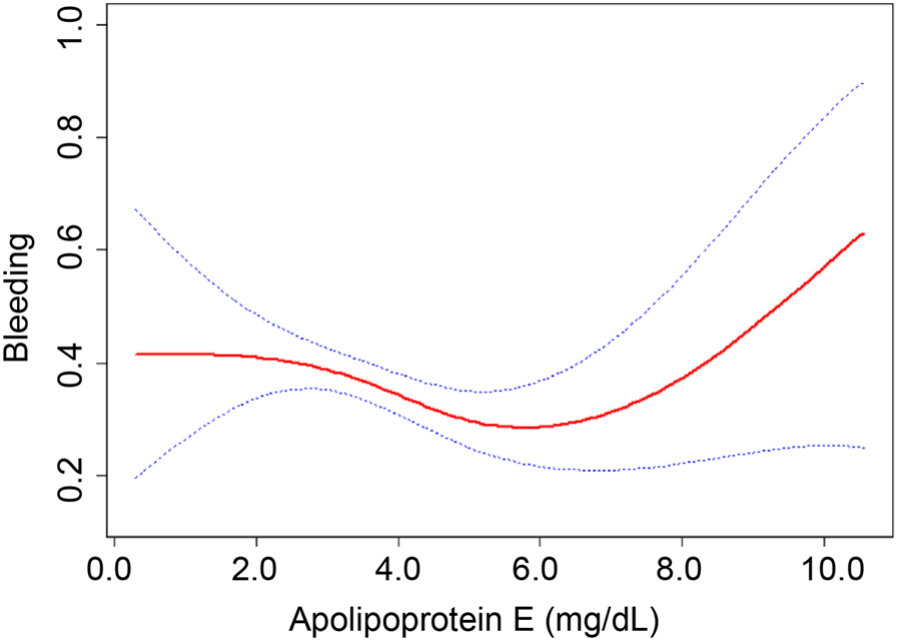
The smooth curve fitting showed the association between the risk of EBB-induced bleeding and plasma ApoE levels after adjusting the relative confounding factors (HDLC, smoking, location of lesion, histological types, stage, and CRP), and factors considered to be clinically relevant, which include sex, age, SBP, DBP, TC, triglyceride, LDLC, diabetes, hypertension, COPD, CHD, ALT, and AST. Dotted lines represented the upper and lower 95% confidence intervals. EBB = endobronchial biopsy; ApoE = apolipoprotein E; HDLC = high density lipoprotein cholesterol; CRP = C-reactive protein; SBP = systolic blood pressure; DBP = diastolic blood pressure; TC = total cholesterol; LDLC = low density lipoprotein cholesterol; COPD = Chronic obstructive pulmonary disease; CHD = coronary heart disease; ALT = alanine aminotransferase; AST = aspartate aminotransferase

Further, we adjusted smoothed plots that suggested a nonlinear relationship between plasma ApoE and the risk of EBB-induced bleeding (Fig. 1). There were threshold effects between plasma ApoE levels and the risk of EBB-induced bleeding in two-piecewise linear regression analysis (Table 4). The risk of EBB-induced bleeding increased with increasing ApoE levels above the turning point (ApoE > 5.9 mg/L) (Table 4; OR, 1.49; 95% CI, 1.03–2.16; *P* < 0.05).

**Table 4:** Threshold effect analysis of ApoE on EBB-induced bleeding using two-piecewise linear regression

Inflection point of ApoE (mg/dL)	Biopsy-induced Bleeding^a^	
	OR (95% CI)	*P* value
Inflection point I		
< 3.5	1.00 (0.70, 1.42) 0.9942	
> 3.5	0.67 (0.47, 0.96) 0.0272	
Inflection point II		
< 5.9	0.82 (0.69, 0.96) 0.0156	
> 5.9	1.49 (1.03, 2.16) 0.0329	

^a^Adjust for: Sex, Age, SBP, DBP, smoking, TC, triglyceride, HDLC, LDLC, location of lesion, histological types, stage, diabetes, hypertension, COPD, CHD, ALT, AST, and CRP

*Apo* apolipoprotein, *HDLC* high density lipoprotein cholesterol, *CRP* C-reactive protein, *SBP* systolic blood pressure, *DBP* diastolic blood pressure, *TC* total cholesterol, *LDLC* low density lipoprotein cholesterol, *COPD* Chronic obstructive pulmonary disease, *CHD* coronary heart disease, *ALT* alanine aminotransferase, *AST* aspartate aminotransferase

## Discussion

To get a clearer understanding of the risks involved in hemorrhage during bronchoscopic biopsies, we studied EBB-induced bleeding in patients with lung cancer. The present study showed that plasma ApoE was associated with EBB-induced bleeding in a non-linear pattern. In order to reduce the risk of biopsy-induced bleeding, especially for massive bleeding during bronchoscopy, it may be appropriate to maintain the levels of plasma ApoE between 3.5 mg/dL and 5.9 mg/dL prior to aggressive biopsy on endobronchial exophytic lesions in lung cancer patients.

Hemorrhage is a very common complication during bronchoscopy, especially when biopsies are performed. It has been noted that malignant lesions are more prone to bleed upon biopsy than benign mucosal lesions [5], and massive blood loss may occur more frequently following EBB [18]. Several risk factors have been proposed to be associated with hemorrhage during bronchoscopy, including immunosuppression, mechanical ventilation, uncontrolled hypertension, severe pulmonary arterial hypertension, thrombocytopenia, anti-coagulant and anti-platelet use, lung transplant, heart failure, liver and kidney disease, and bleeding tendencies [9–11]. However, most of these proposed risk factors are conflicting and lack supporting evidence [12, 13]. To the best of our knowledge, there is no effective indicator available to date for predicting the risk of hemorrhage during bronchoscopy.

ApoE is traditionally an important modulator of many stages of lipoprotein metabolism in the human body. There have also been reports in recent years that ApoE may play a role in some hemorrhagic diseases, such as hypertensive cerebral hemorrhage and subarachnoid hemorrhage [14, 15]. It has been demonstrated that ApoE is associated with an increased risk of amyloid angiopathy and angiorrhexis [16, 17]. It affects the formation of abnormal vessels and pericytes in neurological disorders [14]. Additionally, ApoE impacts the immunomodulatory and oxidative status, which may be associated with poor neurological outcomes after traumatic brain injury and hemorrhage [19, 20].

Our study found that plasma ApoE levels associated well with the incidence of EBB-induced bleeding after adjustment for the main confounding risk factors (HDLC, smoking, location of lesion, histological types, stage, and CRP), or on combined adjustment for factors considered to be clinically relevant (sex, age, SBP, DBP, TC, triglyceride, LDLC, diabetes, hypertension, COPD, CHD, ALT, and AST); the strength of this association did not change. There was a non-linear association between plasma ApoE and the risk of EBB-induced bleeding. We further revealed a piecewise effect of ApoE concentrations. Between 3.5–5.9 mg/dL, ApoE was associated with lower risk of EBB-induced bleeding, whereas higher (> 5.9 mg/dL) concentrations of ApoE were associated with an increased risk of EBB-induced bleeding. Although the mechanism underlying the concentration-dependent relationship between ApoE and the risk of EBB-induced bleeding remains to be elucidated, this finding may be valuable information for the selection and preoperative preparation of biopsy patients.

The present study is the first to reveal the relationship between plasma ApoE and the risk of biopsy-induced bleeding during bronchoscopy. Strengths of this study are, the inclusion of consecutive patients, having fixed bronchoscopists, using only one biopsy maneuver, and a relatively fixed number of biopsies, as well as a relatively fixed ApoE detection time before bronchoscopy. However, some limitations of the current study are worth noting. Firstly, it is still challenging to accurately measure bleeding during bronchoscopy [21]. We could not provide a quantitative measurement of the volume of biopsy-induced bleeding in our study. Patients were divided into a bleeding group or a non-bleeding group based only on whether they received hemostasis during EBB. Therefore, this classification may not accurately group patients with minimal bleeding. Secondly, it is well known that ApoE has three isoforms in humans, namely ApoE2, ApoE3 and ApoE4 [20]. We however did not do any further genotyping of ApoE because information on these variables was unavailable in the retrospective data. Therefore, the results of this study may only be representative of Asian lung cancer population in East China.

## Conclusions

In conclusion, our study showed that plasma ApoE is associated with the risk of EBB-induced bleeding in patients with lung cancer in a non-linear pattern, and the relative safe levels of ApoE may be 3.5–5.9 mg/dL. Our findings highlight the importance of ApoE levels in bleeding during EBB and may have implications for risk assessment and risk modification prior to bronchoscopy. However, future prospective studies involving a larger number of subjects and basic research are warranted to fully evaluate its clinical value and the mechanisms involved.

## Abbreviations

ALT: Alanine aminotransferase
APC: Argon plasma coagulation
ApoE: Apolipoprotein E
APTT: Activated partial thromboplastin time
AST: Aspartate aminotransferase
CHD: Coronary heart disease
CI: Confidence interval
COPD: Chronic obstructive pulmonary disease
CRP: C-Reactive protein
DBP: Diastolic blood pressure
EBB: Endobronchial biopsy
HDLC: High density lipoprotein cholesterol
LDLC: Low density lipoprotein cholesterol
LMA: Laryngeal mask airway
OR: Odds ratio
PT: Prothrombin time
SBP: Systolic blood pressure
TC: Total cholesterol
WBC: White blood cell
